# Medullary Thyroid Carcinoma: Do Ultrasonography and F-DOPA-PET-CT Influence the Initial Surgical Strategy?

**DOI:** 10.1245/s10434-018-6829-3

**Published:** 2018-10-10

**Authors:** Lindsay Brammen, Martin B. Niederle, Philipp Riss, Christian Scheuba, Andreas Selberherr, Georgios Karanikas, Gerd Bodner, Oskar Koperek, Bruno Niederle

**Affiliations:** 1 Section “Endocrine Surgery”, Division of General Surgery, Department of Surgery, Medical University Vienna, Vienna, Austria; 2 Department of Anesthesiology, Medical University Vienna, Vienna, Austria; 3 Department of Biomedical Imaging and Image-Guided Therapy, Division of Nuclear Medicine, Medical University Vienna, Vienna, Austria; 4 Department of Biomedical Imaging and Image-Guided Therapy, Division of Radiology, Medical University Vienna, Vienna, Austria; 5 Department of Pathology, Medical University Vienna, Vienna, Austria; 6 Former Chief of the Section “Endocrine Surgery”, Department of Surgery, Medical University Vienna, Vienna, Austria

## Abstract

**Background:**

At the time of diagnosis, one-third of medullary thyroid carcinoma (MTC) patients show lymph node (LN) or distant metastasis. A metastasized MTC requires different surgical strategies.

**Objective:**

This study aimed to determine the value of ultrasound and [18F]fluoro-dihydroxyphenylalanine positron emission tomography with computed tomography (F-DOPA-PET-CT) in localizing MTC, as well as LN and distant metastasis.

**Methods:**

The study included 50 patients (24 males/26 females) with preoperative ultrasound, F-DOPA-PET-CT, and histologically proven MTC. Imaging results were correlated with both preoperative basal calcitonin (bCt) levels and final histology.

**Results:**

Tumors were classified as pT1a:17 (diameter, mean ± standard deviation: 5.8 ± 3.0 mm), pT1b:15 (15.0 ± 3.2 mm), pT2:9 (27.3 ± 7.0 mm), and pT3:9 (38.3 ± 24.2 mm). The median bCt level was 202 pg/mL (lower/upper quartile: 82/1074 pg/mL). Ultrasound was positive for tumor in 45/50 (92%) patients (20.0 ± 16.0 mm) and negative in 5 patients (3.2 ± 2.2 mm). Overall, 43/50 (86%) patients had positive F-DOPA local scans (20.0 ± 16.4 mm), while 7 (14%) patients were negative (7.7 ± 8.1 mm). Lastly, 21/50 (42%) patients had LN metastasis; 8/21 (38%) patients had positive LNs suspected with ultrasound, and 12/21 (57%) patients had positive LNs suspected with F-DOPA. Tumor and LN sensitivity of ultrasound was 92% and 43%, respectively, and 86% and 57% of F-DOPA-PET-CT, respectively. In 3/50 (6%) patients and 3/50 (6%) patients, mediastinal LN metastasis and distant metastasis, respectively, were diagnosed only by F-DOPA-PET-CT.

**Conclusion:**

Ultrasound and F-DOPA-PET-CT are sensitive for the localization of MTC but not for the presence and location of LN metastasis (limitations: size/number). Only F-DOPA ensures the diagnosis of distant metastasis and influences the extent of LN surgery. Surgical strategy cannot be predicted based on neither ultrasound nor F-DOPA-PET-CT.

In medullary thyroid carcinoma (MTC), the number of positive lymph nodes (LNs) and involved anatomical compartments are cancer-specific prognostic factors.^1^ Exact preoperative staging may help to select a stage-adapted surgical strategy.

Neck ultrasound is the first imaging modality used in MTC to assess the local extent and regional lymphatic spread. MTC as a neuroendocrine tumor is able to absorb, store, and decarboxylate dopamine. Therefore [18F]fluoro-dihydroxyphenylalanine positron emission tomography with computed tomography (F-DOPA-PET-CT), combining anatomic and ‘functional’ imaging, may further improve initial local and distal staging of biochemically suspected MTC.

F-DOPA-PET-CT is one of the recommended imaging modalities in patients with persistent MTC and basal calcitonin (bCt) levels ≥ 150 pg/mL before reoperations;^2^–^5^ however, no studies with a larger patient population evaluating ultrasound in addition to F-DOPA-PET-CT in locating the primary tumor, LN and distant metastasis, and possible consequences to the surgical strategy, have been performed.

This prospective observation study aims to determine the value of ultrasound and F-DOPA-PET-CT in radiomorphological detection of primary tumors, identification of possible LN metastasis, and detection of possible distant metastasis before initial surgery, and how a combination of these techniques may help to improve the standardized diagnostic and therapeutic protocol correlating preoperative biochemical and postoperative morphological results.

## Materials and Methods

### Patients

Overall, 50 patients (24 males/26 females) with biochemical criteria of MTC^6^ who underwent initial surgery were included in the study. Inclusion criteria consisted of preoperative neck ultrasound and F-DOPA-PET-CT, biochemical parameters, surgery, final histology, and follow-up in all patients.

The present study was approved by the local Institutional Review Board (EK reference number: 1083/2016). All patients signed written informed consent for all diagnostic and therapeutic procedures.

### Laboratory Measurements

Since 1994, in our institution, the consequent determination of calcitonin (‘calcitonin screening’) has been performed during the diagnostic work-up of all thyroid nodules regardless of thyroid function, sonomorphology, and size of the thyroid lesion before surgery.

Following the local diagnostic standard operating procedure (SOP), bCt > 8 pg/mL for males or > 6 pg/mL for females, *and* calcium-stimulated Ct > 100 pg/mL, were considered highly suspicious of MTC and were therefore an indication for surgery.

The Ct levels were assessed using a two-site chemiluminescent immunometric assay from Diagnostic Products Corporation (Los Angeles, CA, USA), running as a fully automated test on a Siemens Immulite 2000 Immunoassay System (Siemens Diagnostics) with a lower detection limit of 0.5 pg/mL and an intra-assay coefficient variation of ≤ 2%.

### Diagnostic Procedures

#### Neck Ultrasound

A standardized neck ultrasound of the cervical and supraclavicular regions (General Electric [GE] LOGIQ E9 device; 9 MHz probe) was performed and analyzed preoperatively by one radiologist (GB), who was aware of the biochemically supposed diagnosis. A suspicious local lesion was characterized as a solid hypoechoic, microcalcification, echogenic foci, intranodular vessel and, if advanced disease, extension beyond the thyroid margin.^7^ Positive metastatic LNs were described as round in shape, necrotic, calcified, loss of the nodal hilum, and peripheral vascularity.^8^

### F-DOPA-PET-CT

As previously described,^9^ PET/CT in a two-dimensional mode with subsequent reconstruction from the top of the skull to the thigh using an integrated camera was performed. One experienced nuclear medicine physician (GK) and one experienced radiologist (GB) interpreted all imaging examinations independently. A lesion was considered ‘PET positive’ if focal was greater than physiologic tracer uptake and greater than background blood-pool activity. LNs were considered highly suspect for malignancy when the diameter was > 10 mm, and showed round configuration, contrast enhancement, and F-DOPA enhancement without fatty hilus.^3^

True-positive patients had at least one pathologically verified malignant lesion, whereas false-negative patients had positive histopathological results but negative F-DOPA-PET-CT findings.

#### Surgical Procedure

In accordance with our surgical SOP (6), all patients underwent (total) thyroidectomy with bilateral central neck dissection (level VI) and bilateral lateral lymphadenectomy (level II–V) independent of the bCt levels, sparing all vessels, muscles and nerves (functional lateral neck dissection). The surgeon knew all the results of preoperative ultrasound and F-DOPA-PET-CT imaging. Only the knowledge of F-DOPA-PET-CT-positive LNs in the mediastinum and distant metastasis influenced the extent of surgery. In patients with positive F-DOPA-PET-CT imaging in the mediastinum, but negative imaging for distant metastasis, a mediastinal LN dissection was conducted. In patients with distant metastasis, a radical central, but a less radical lateral, cervical LN dissection was conducted to avoid complications by positive residual LNs in the central neck.

Surgical outcome was based on the assessment of bCt (and calcium-stimulated Ct) levels determined during the follow-up program. A biochemical cure was achieved if the postoperative b and stimulated Ct levels were undetectable.

#### Pathological Examinations

The pathologist was aware of the elevated bCt levels, and therefore of the suspected MTC. Macroscopically suspected lesions (primary tumor; LNs) were stained with hematoxylin and eosin (H&E), and immunohistochemistry was performed by one pathologist (OK) to confirm primary or metastatic MTC.

#### TNM Classification

TNM classification based on the 7th Edition of the American Joint Committee on Cancer Staging Manual was used for all histopathological results.^10^

### Statistical Analysis

Descriptive statistics analyzed age, sex, tumor characteristics (diameter), number of LNs detected and surgically removed, and bCt levels. Data are given as median (25th–75th percentile) or mean (± standard deviation). Sensitivity, specificity, positive predictive value (PPV), and negative predictive value (NPV) were calculated applying Statistical Package for Social Sciences (SPSS) version 23 for Windows (IBM Corporation, Armonk, NY, USA).

Sensitivity for ultrasound and F-DOPA-PET-CT was calculated for different tumor sizes (pT) and different bCt cut-offs. Patients were grouped into those with tumors ≤ 10 mm (‘microcarcinoma’; pT1a) and those with tumors > 11 mm (‘macrocarcinoma’; pT1b–T4).

Patients with a bCt cut-off level > 43 pg/mL have MTC independent of the patient’s sex.^11^ The American Thyroid Association (ATA) guidelines recommended functional imaging techniques in patients with bCt > 150 pg/mL, to localize persistent disease.^12^

The optimal cut-off for bCt in diagnosing lateral compartment LN metastasis was calculated using a receiver operating characteristic (ROC) curve (not shown in detail). Positive lateral LNs were only found in patients with bCt levels ≥ 85 pg/mL (sensitivity 100%). Sensitivity, specificity, positive predictive and NPVs to diagnose and locate positive lateral LNs were calculated based on this bCt cut-off.

## Results

Patient characteristics, correlation of tumor classification, and imaging results are summarized in Table 1. Tables 2 and 3 provide the overall results, localizing the primary tumor and affected LNs by ultrasound and F-DOPA-PET-CT.

**Table 1:** Patient characteristics

No. of patients		50
Males:females		24:26
Mean age, years (range)		56.9 (8.8–78.9)
Basal CT^a^ (range)		202 pg/mL (82–1074)
CEA (range)		12.8 µg/mL (3.85–52.3)
T-stage classification^b^ [*n* (%)]		
pT1a		17/50 (34)
pT1b		15/50 (30)
pT2		9/50 (18)
pT3		9/50 (18)
Multifocal [*n* (%)]		8/50 (16)
Unilateral versus bilateral		2 versus 6
Hereditary MTC [*n* (%)]		8/50 (16)
Mean tumor size [mm (± SD)]		18.3 (± 16.0)
N1^c^ [*n* (%)]		21/50 (42)
N1a [*n* (%)]	Central	18/21 (85.7)
N1b [*n* (%)]	Lateral	16/21 (76.2)
	Mediastinal	3/21 (14)
Mean number of LNs removed (± SD)		74 (± 7)
Mean number of positive LNs (± SD)		6.7 (± 2)
M1^d^ (%)		3/50 (6)

*CEA* Carcinoembryonic antigen, *MTC* medullary thyroid carcinoma, *SD* standard deviation, *LNs* lymph nodes, *UICC* Union for International Cancer Control, *LNs* lymph nodes

^a^Calcitonin (Siemens Diagnostic Products Corporation). Normal range: bCt: ≤ 8 pg/mL males, ≤ 5 pg/mL females; CEA: 0–3.8 μg/L (0–6.5 μg/L in smokers)

^b^UICC 2010^10^ (*p*: pathological classification). pT1a: ≤ 10 mm; pT1b: 11–20 mm; pT2: 21–40 mm; pT3: > 40 mm or any size with minimal extrathyroidal extension; pT4: moderately advanced or very advanced disease

^c^N1: regional LN metastasis; N1a: metastasis to level VI (pretracheal, paratracheal, and prelaryngeal/Delphian LNs N1b. Metastasis to unilateral, bilateral, or contralateral cervical (levels I, II, III, IV, or V), retropharyngeal or superior mediastinal LNs (level VII)

^d^M1: distant metastasis

**Table 2:** Diagnosis of primary tumor: ultrasound and F-DOPA-PET-CT

	Ultrasound			F-DOPA-PET-CT		
	Yes (*n*)	No (*n*)	Sensitivity (%)	Yes (*n*)	No (*n*)	Sensitivity (%)
All (pT1a–pT3)	45	5	90	43	7	86
pT1a	12	5	71	11	6	65
pT1b	15	0	100	15	0	100
pT2	9	0	100	8	1	89
pT3	9	0	100	9	0	100
pT1a (maxDM ≤ 10 mm)	12	5	71	11	6	67
pT1b–4 (maxDM > 10 mm)	32	0	100	31	1	97
Calcitonin level^a^						
bCt ≤ 43 pg/mL	8	1	89	4	5	44
bCt > 43 pg/mL^b^	41	0	100	39	2	95
bCt ≤ 150 pg/mL^c^	22	1	96	16	7	70
bCt > 150 pg/mL	27	0	100	27	0	100

*F*-*DOPA*-*PET*-*CT* [18F]fluoro-dihydroxyphenylalanine positron emission tomography with computed tomography, *pT* pathological tumor classification (11), *maxDM* maximal tumor diameter, *bCt* basal calcitonin level, *MTC* medullary thyroid carcinoma

^a^Two-site chemiluminescent immunometric assay: Diagnostic Products Corporation (Los Angeles, CA, USA)

^b^All patients with bCt > 43 pg/mL were shown to have MTC, independent of the patient’s sex

^c^bCT of 150 pg/mL is the lowest value recommended in the American Thyroid Association guidelines for performing functional imaging techniques in (persisting) MTC

**Table 3:** Value of ultrasound and F-DOPA-PET-CT: analysis of patients with central and lateral neck LN metastasis

Lymph node metastasis	Ultrasound			F-DOPA-PET-CT		
	Central	Lateral	Overall^a^	Central	Lateral	Overall^a^
True positive (*n*)	1	9	9	5^b^	12	12
True negative (*n*)	32	33	28	32	34	29
False positive (*n*)	0	1	1	0	0	0
False negative (*n*)	17	7	12	13	4	9
Sensitivity (%)	6	56	43	28	75	57
Specificity (%)	100	97	97	100	100	100
PPV (%)	100	90	90	100	100	100
NPV (%)	65	83	70	71	89	76
Accuracy (%)	66	84	74	74	92	82

*F*-*DOPA*-*PET*-*CT* [18F]fluoro-dihydroxyphenylalanine positron emission tomography with computed tomography, *LN* lymph node, *PPV* positive predictive value, *NPV* negative predictive value

^a^Evaluation of ALL neck compartments together

^b^Patients had both positive central and lateral LNs

### Primary Tumor

Neck ultrasound was true positive in 45/50 (90%) patients (mean diameter: 20.0 ± 16.0 mm). In 5 (10%) patients (3.2 ± 2.2 mm; 4/5 with bilateral thyroid nodules), the specific malignant nodule was not preoperatively identified in the correct lobe (Table 3).

F-DOPA-PET-CT was true positive in 43/50 (86%) patients (20.0 ± 16.4 mm). When studying the F-DOPA scans for diagnosis of the primary tumors, the preoperative diagnostic scans were false negative in 7 (14%) patients (7.7 ± 8.1 mm; 3/7 with bilateral thyroid nodules) (Table 2).

### Lymph Node Metastasis

LN metastasis was pathologically reported in 21/50 (42%) patients. Ultrasound and F-DOPA-PET-CT predicted true-positive LN metastasis in 9/21 (42.9%) patients and 12/21 (57.1%) patients, respectively. The overall sensitivities of ultrasound and F-DOPA-PET-CT were 43% and 57%, respectively (Table 3).

### Compartment Correlation

#### Central Neck (N1a; Localized Disease)

In terms of diagnosing central LN metastasis, neck ultrasound was true positive in one patient (diameter: 20 mm), while 17/18 (94.4%) patients were false negative (6.2 ± 4.87 mm; sensitivity: 6%; specificity: 100%; overall accuracy: 66%) (Table 3).

F-DOPA-PET–CT was true positive in 5/18 (27.7%) patients (15.0 ± 5.77 mm), and false negative in 13/18 (72.2%) patients (4.41 ± 2.65 mm; sensitivity: 28%; specificity: 100%; overall accuracy: 74%).

#### Lateral Neck and Mediastinum (N1b; Regional Disease)

LN metastasis in the lateral neck was found in 16/21 (76.2%) patients; neck ultrasound was true positive in 9/16 (56%) patients (11.88 ± 13.31 mm). With one false-positive and seven false-negative patients (6.93 ± 6.56 mm), a sensitivity of 56% and specificity of 97% was calculated (overall accuracy: 84%).

In F-DOPA-PET-CT, 12/16 (75%) scans were true positive (11.08 ± 12.08 mm); four (25%) were false negative (5.62 ± 5.09 mm). No patients were false positive (sensitivity: 75%; specificity: 100%; overall accuracy: 92%).

#### Upper Mediastinum

Additionally, F-DOPA-PET-CT was true positive in three patients with mediastinal LN metastasis. These patients had bCt levels of 12,411, 14,360, and 14,686 pg/mL, respectively, and none of these patients had distant metastases.

### Comparing the Diagnostic Value of bCt and Imaging Techniques

All 16 patients with LN metastasis in the lateral neck had bCt levels of > 85 pg/mL (sensitivity: 100%); however, given that 21 LN-negative patients had bCt levels > 85 pg/mL, the specificity was 38% (Table 4).

**Table 4:** Biochemical calculation (optimal bCT cut-off for lateral LN metastasis: > 85 pg/mL) and radiological methods (ultrasound or F-DOPA-PET-CT) to predict lateral neck LN metastasis

Lymph node metastasis in the lateral neck^a^	bCt cut-off > 85 pg/mL^b^	Ultrasound	F-DOPA-PET-CT
True positive (*n*)	16	9	12
True negative (*n*)	13	33	34
False positive (*n*)	21	1	0
False negative (*n*)	0	7^a^	4^b^
Sensitivity (%)	100	56	75
Specificity (%)	38	97	100
PPV (%)	43	90	100
NPV (%)	100	83	89
Accuracy (%)	58	84	92

*F*-*DOPA*-*PET*-*CT* [18F]fluoro-dihydroxyphenylalanine positron emission tomography with computed tomography, *bCt* basal calcitonin level, *LN* lymph node, *PPV* positive predictive value, *NPV* negative predictive value

^a^Patients had 1–17 positive lateral LNs

^b^Lateral lymph node metastasis was only found in patients with bCt levels ≥ 85 pg/mL

### Number of Metastasis, Sensitivity of Ultrasound and F-DOPA-PET-CT

The sensitivity for each imaging technique was analyzed for patients with less than (*n* = 11) or more than 10 (*n* = 8) LN metastasis. The sensitivity of ultrasound or F-DOPA-PET-CT in patients with ≤ 10 positive LNs was 9% and 27%, respectively, and 80% and 90%, respectively, for patients with > 10 positive LNs. Analyzing the individual patients with true-positive F-DOPA-PET-CT and more than 10 positive LNs, the positive LNs were arranged as LN bulks.

### Distant Disease

Three patients (two males, one female; mean age 63.1 years) had distant metastasis diagnosed in F-DOPA-PET-CT preoperatively (liver metastasis: *n* = 2; bCt values: 1942 pg/mL and 22,953 pg/mL; lung, liver and bone metastasis: *n* = 1; bCt 8397 pg/mL). All three patients underwent a palliative surgical approach with thyroidectomy, central neck, and a less extended lateral LN dissection. F-DOPA-PET-CT positively identified all distant metastasis.

### Morbidity

No permanent complications (hypoparathyroidism, paralysis of the recurrent nerve) were documented in the study patients. Temporary hypoparathyroidism was documented in 7/50 (14%) patients, and temporary paralysis was documented in 4/100 (4%) nerves at risk. Two patients suffered from unilateral temporary weakness of the accessory nerve and shoulder dysfunction.

### Follow-Up

Table 5 summarizes the biochemical follow-up of the study population. By definition, 34/50 (68%) patients were cured. In one (4.8%) patient and in 15 (30%) patients, recurrent and persisting disease, respectively, were documented. Five (4.8%) patients died of progressive MTC.

**Table 5:** Staging and follow-up of the study population (mean follow-up: 60 ± 31 months)

Stage	T	N	M	Number of patients			
				Cured^a^	Persisting disease^b^	Recurrent disease^c^	Total
I	1a	0	0	14			14
	1b			7			7
II	2	0	0	4		1	5
	3			3			3
III	1a	1a	0	2			2
	1b			1			1
	2			1			1
	3						
IVA	4a	0/1a					
	1a	1b	0	1			1
	1b			1	6		7
	2				1		1
	3				5 (died: 2^d^)		5
IVB	4b	Any N	1				
IVC	Any T	Any N			3 (died: 2^d^)		
			*n*	34 (68%)	15 (30%; died: 4.8%^d^)	1	50

TNM, see Edge and Compton^10^ and Wells et al.^12^

*bCt* Basal calcitonin level

^a^Cured: At the time of follow-up, normal/undetectable bCt levels

^b^Persisting disease: bCt levels never decreased to normal/undetectable values

^c^Recurrent disease: Normalization of bCt levels for at least 1 year, than a recurrent increase

^d^*n* patients died of MTC

## Discussion

This study analyses the value of ultrasound and F-DOPA-PET-CT in localizing MTC, and LN and distant metastasis, before initial surgery to select stage-adapted surgery.

Neck ultrasound and F-DOPA-PET-CT achieved an overall sensitivity of 90% and 86%, respectively, in locating MTC in the correct thyroid lobe. As expected, the sensitivity depends on the size of the tumor and whether it is found as a solitary nodule or in multiple nodules. When localizing microcarcinoma (tumor size ≤ 10 mm), the sensitivity for ultrasound and F-DOPA-PET-CT was 71% and 67%, respectively, and 100% and 97%, respectively, for macrocarcinoma (tumor size > 10 mm). In four patients with multinodular goiter, ultrasound failed to diagnose the malignant micronodules in all patients, while F-DOPA-PET-CT failed in three of the four patients. In the literature, the sensitivity for ultrasound to localize the primary tumor ranges between 27 and 97%, and the sensitivity of F-DOPA-PET-CT ranges between 65 and 100%.^22^–^26^ This current study exhibited high sensitivity in lesion-based ultrasound and F-DOPA-PET-CT. Ni Mhuircheartaigh et al.^13^ reported an average nodule size of at least 15.6 mm diagnosed in PET-CT. Therefore, size seems an unreliable criterion when it comes to localizing a malignant thyroid tumor.^14^–^16^ False negatives could also be due to the fact that F-DOPA is not retained for a long period of time in small-sized MTCs.^2^,^17^

Several studies document that tumor burden positively correlates with bCt levels.^13^,^14^ In patients with bCt > 43 pg/mL and > 150 pg/mL, a sensitivity of 100% was achieved for diagnosing the primary tumor with ultrasound. However, one must take into account that the radiologist may have been biased in the preoperative neck ultrasound examinations, considering that all patients sent for ultrasound met the biochemical criteria for MTC. In addition, the experience of the radiologist also plays a role in the correct diagnosis. F-DOPA-PET-CT for primary tumor showed a sensitivity of 95% in patients with bCt > 43 pg/mL, and 100% in patients with bCt > 150 pg/mL, compared with 44% and 70%, respectively, in patients with lower bCt levels. Therefore, bCt levels influence the sensitivity of F-DOPA-PET-CT in primary tumor localization.

There is a broad overlap in the aspect of benign and malignant LNs. In this study, a low sensitivity was achieved in diagnosing LN metastasis by ultrasound. While the overall sensitivity of 43% lies within the reported range (29–100%^18^–^31^), it is still much lower than the pooled sensitivity in the reviewed literature (72%). In terms of diagnosing central LN metastasis, neck ultrasound demonstrated only a 6% sensitivity. In particular, micrometastasis may be hidden by the thyroid gland and may therefore be missed on ultrasound. However, this study demonstrated a high PPV (100%), higher than previous studies (77–83%^24^,^32^–^34^).

Ultrasound demonstrated a sensitivity of 56% and specificity of 97% in diagnosing lateral LN metastasis. This study demonstrated a high PPV (90%), similar to previous studies (85–88%^24^,^32^,^35^). Overall accuracy in the central compartment (66%) was lower than in the lateral compartment (84%), in accordance with other studies.^29^,^36^,^37^

When analyzing the individual LN compartments, F-DOPA-PET-CT exhibited only 28% sensitivity and 100% specificity for the central compartment. A higher sensitivity (75%) and specificity (100%) were seen in the lateral compartment. When applying F-DOPA-PET-CT in diagnosing and localizing LN metastasis, an overall sensitivity of 57% was demonstrated. In patients with bCt > 43 pg/mL and > 150 pg/mL, a sensitivity of 60% and 73%, respectively, was achieved. As previously mentioned, the small size of LN metastasis and the short retention time of F-DOPA may affect LN imaging and therefore the low overall sensitivity.

As shown by Machens and Dralle^1^, surgical cure is possible in patients with ≤ 10 LN metastases. A subanalysis demonstrated a very low sensitivity of 27% in localizing positive nodes by F-DOPA-PET-CT when applying the cut-off of ≤ 10 LN metastases. This sensitivity increased to 90% in patients with ≥ 10 LN metastases. Therefore, in patients where only a few, or micro, LN metastases are expected, F-DOPA-PET-CT is not beneficial for diagnostic purposes. Therefore, these findings justify a radical surgical procedure, even in the lateral neck, taking into account possible skip lesions.^1^

A limitation of ultrasound is that LN metastasis in the mediastinum or distant metastasis cannot be diagnosed; however, F-DOPA-PET-CT demonstrated 100% sensitivity and specificity in diagnosing positive mediastinal LNs and distant metastasis. According to the current ATA guidelines,^12^ patients with Ct levels > 500 pg/mL before the initial operation, or patients with Ct levels > 150 pg/mL in follow-up should undergo CT imaging with/without PET.^12^,^38^ All patients with positive LNs in the mediastinum and/or with distant metastasis had bCt values ≥ 500 pg/mL. Given the high sensitivity of F-DOPA-PET-CT with mediastinal LNs and distant metastases, all patients with bCt values > 500 pg/mL should primarily undergo F-DOPA-PET-CT to diagnose mediastinal LNs or distant metastasis preoperatively.

Patients with positive LNs in the upper mediastinum, but without distant metastasis, may benefit from mediastinal dissection. Furthermore, in patients with distant metastasis, a less extended neck surgery should be considered to avoid local complications and to circumvent complications arising from an extended surgery in the lateral neck.

Generally, the primary workup by ultrasound documents a nodule that is possibly malignant with a clear indication for surgery. F-DOPA-PET-CT improves the results in the central neck from a sensitivity of 6% to 28% in respect of diagnosing positive central nodes. However, neither ultrasound nor F-DOPA-PET are helpful in limiting surgery in the central neck because of the overall imprecise radiological localization of the primary tumor and LN metastasis.

Independent of a positive or negative preoperative imaging study, the primary operation has to meet oncological standards ([total] thyroidectomy; bilateral central [level VI] dissection) in patients who fulfill the biochemical criteria for MTC. One has to keep in mind that the study was performed within a ‘calcitonin screening program’ documenting pT1a in 17/50 (34%) patients (diameter ≤ 5 mm in 6 [25%] patients), 3 (17.6%) of whom presented with only one or two central LN micrometastases.

Micrometastatic changes in LNs may also influence the low sensitivity in correctly diagnosing and localizing lateral LN metastasis. While the PPV was only 38%, retrospectively, it may be recommended to apply ‘functional lateral neck dissection’ in at least all patients with bCt > 85 pg/mL. However, in 21/50 (42%) patients, the lateral neck dissection revealed negative LNs.

When determining which preoperative examinations to conduct, the advantages and disadvantages for each test must be evaluated. Neck ultrasound is non-invasive, widely available, less expensive than other imaging techniques, and is very safe as no ionizing radiation is used; however, the results are dependent on the radiologist and his/her training and experience. F-DOPA-PET-CT is a functional technique able to provide important anatomical information, especially when it comes to diagnosing distant metastasis. However, its disadvantages include limited availability, high radiation dose (approximately 25 mSv^39^,^40^ depending on body weight), possible allergic reactions to the contrast medium, and it is expensive.

## Conclusions

This large, single-center study evaluated how neck ultrasound and whole-body F-DOPA-PET-CT may influence the initial surgical strategy in a center with routine measurement of calcitonin in all patients with thyroid nodules. While the sensitivity of ultrasound in diagnosing the primary tumor was very high (92%), it was unfortunately quite low (43%) in detecting the presence and location of LN metastasis. F-DOPA PET-CT also exhibited a good sensitivity of 86% in detecting the primary tumor, but also a low sensitivity (57%) when diagnosing the presence and location of LN metastasis. Therefore, the extent of lateral LN surgery cannot be determined, based on neither ultrasound nor F-DOPA-PET-CT. However, the detection of distant metastasis by functioning imaging may help limit the extent of lateral neck and/or mediastinal dissection. Patients who fulfill the biochemical criteria for MTC should undergo a tailored surgical approach.
